# Optical coherence tomography for observing development of pulmonary arterial vasa vasorum after bidirectional cavopulmonary connection in children

**DOI:** 10.1371/journal.pone.0215146

**Published:** 2019-04-08

**Authors:** Yasunobu Hayabuchi, Yukako Homma, Shoji Kagami

**Affiliations:** Department of Pediatrics, Tokushima University, Kuramoto-cho-3, Tokushima, Japan; University of Sydney, AUSTRALIA

## Abstract

**Background:**

Hypoxia and low pulmonary arterial (PA) blood flow stimulate the development of systemic-to-pulmonary collateral blood vessels, which can be an adverse factor when performing the Fontan operation. The aim of this study was to use optical coherence tomography (OCT) to elucidate the morphological changes in PA vasculature after creation of a bidirectional cavopulmonary connection (BCPC) in children.

**Methods:**

This prospective study evaluated PA wall thickness and development of PA vasa vasorum (VV) in the distal PA of eight patients (BCPC group, 1.3 ± 0.3 years) and 20 age-matched children with normal pulmonary artery hemodynamics and morphology (Control group, 1.4 ± 0.3 years). VV development was defined by the VV area ratio, defined as the VV area divided by the adventitial area in cross-sectional images.

**Results:**

There was no significant difference in PA wall thickness between the BCPC and control groups (0.12 ± 0.03 mm vs. 0.12 ± 0.02 mm, respectively). The VV area ratio was significantly greater in the BCPC group than in the Control group (14.5 ± 3.5% vs. 5.3 ± 1.6%, respectively; p<0.0001).

**Conclusion:**

OCT is a promising new tool for evaluating PA pathology, including the development of VV in patients after BCPC.

## Introduction

The vasa vasorum (VV) in the pulmonary circulation are the microcirculatory network of the systemic circulation and, similar to their role in systemic vessels, are thought to contribute to vascular integrity through supply of oxygen and nutrients to the outer part of the vessel wall [1,2]. Low pulmonary arterial (PA) flow stimulates angiogenesis in the systemic circulation and increases systemic-to-pulmonary arterial (SPCA) blood flow via remodeling and proliferation of the SPCA network [2–4]. Hypoxia-induced PA VV expansion has also been described in relation to vascular remodeling and angiogenesis in the pulmonary circulation [4]. Due to these mechanisms, SPCA frequently develop after creation of bidirectional cavopulmonary connections (BCPC), and can be deleterious at the time of a subsequent Fontan procedure due to the resultant increase in PA blood flow [5,6]. Hence, evaluation of the PA vasculature before performing the Fontan procedure is important in such children.

However, the current understanding of the pathophysiology of the PA is incomplete, since information regarding alterations in pulmonary vasculature is primarily obtained from autopsy or tissue specimens. Only limited data related to intravascular imaging of the PA is available, primarily due to a lack of adequate imaging techniques. The technique of optical coherence tomography (OCT) has led to significant advancements in intravascular imaging, which has enabled precise examination of PA morphology. Recent reports have shown that OCT is useful not only for the diagnosis of pulmonary hypertension, but also for evaluation of the response to treatment [7,8].

The present study evaluated morphological changes in the PA, including the development of PA VV, to elucidate pathological changes in the PA of children in the interim between creation of BCPCs and the Fontan operation.

## Materials and methods

### Patients characteristics

This was a single-center, prospective, observational study. The study group included 8 consecutive patients after BCPC (BCPC group; mean age, 1.3 ± 0.3 y). The diagnoses in them included: single ventricle (n = 4), pulmonary atresia with intact ventricular septum (n = 3), and double outlet right ventricle (n = 1). The patients underwent cardiac catheterization for evaluation prior to the Fontan procedure. Twenty age-matched children with normal pulmonary artery morphology and pressure were also enrolled (control group; age, 1.4 ± 0.3 y). The control group consisted of patients with diagnoses as follows: nine patients after recovery from Kawasaki disease without any coronary artery stenosis or myocardial ischemia, nine patients with patent ductus arteriosus with a pulmonary to systemic blood flow (Qp/Qs) ratio of less than 1.1 who were scheduled to undergo catheter occlusion, and two patients with ventricular septal defect associated with aortic regurgitation whose Qp/Qs was less than 1.1. Participants were included in this study only if they were between 1 and 2 years of age between December 2013 and August 2016. All patients had been scheduled for evaluation of their circulatory status. All protocols were approved by the Institutional Review Board of Tokushima University Hospital and conformed to the ethical guidelines of the Declaration of Helsinki (1975). The parents of all subjects provided their written, informed consent for their children to participate in the study.

### OCT image recordings

Catheterization and angiography using an Integris Allura 9 Biplane cardiac catheterization system (Phillips Medical Systems, Best, The Netherlands) were performed using 4 to 6 Fr catheters. All patients were examined under general anesthesia and endotracheal intubation. No patient suffered complications from conventional angiography and OCT imaging. OCT image acquisition was performed using a commercially available ILUMIEN FD-OCT Imaging System (St. Jude Medical Japan Co. Ltd., Tokyo, Japan). Images were obtained from a segmental PA of the inferior lobe both in the stationary state and during a pull-back rate of 54 mm/sec. The low-molecular-weight dextran flush technique was used to run off blood and obtain high quality images.

We evaluated the PA wall thickness and expansion of the PA VV in distal pulmonary vessels with a diameter of 2.0–2.5 mm, because our previous investigation demonstrated that this size of PA was most frequently delineated [9]. Development of the VV was indicated by the VV area ratio, defined as the VV area divided by the adventitial area in cross-sectional images. Area calculation was evaluated with image analysis software (ImageJ 1.45, National Institutes of Health, Bethesda, Maryland, U.S.A.). Since the outer boundary of the adventitia was unclear, it was defined as 500 μm outside the intima-media layer so the adventitia area could be calculated. All the morphometric evaluations were performed offline on saved images. Finally, each subject's parameters were determined by selecting and averaging 5 cross section.

### Statistical analysis

All data are expressed as means (standard deviation) (SD) or as medians with the 5th– 95th percentiles. Statistical significance was determined using Student’s t-test or Mann-Whitney’s *U*-test, as appropriate. All statistical data were calculated using Prism version 6.0 (GraphPad Software, San Diego, CA, USA) and JMP 11 (SAS Institute, Inc., Cary, NC, USA) software installed on a desktop computer. A value of p < 0.05 (two-sided) was considered significant.

## Results

**Fig 1:**
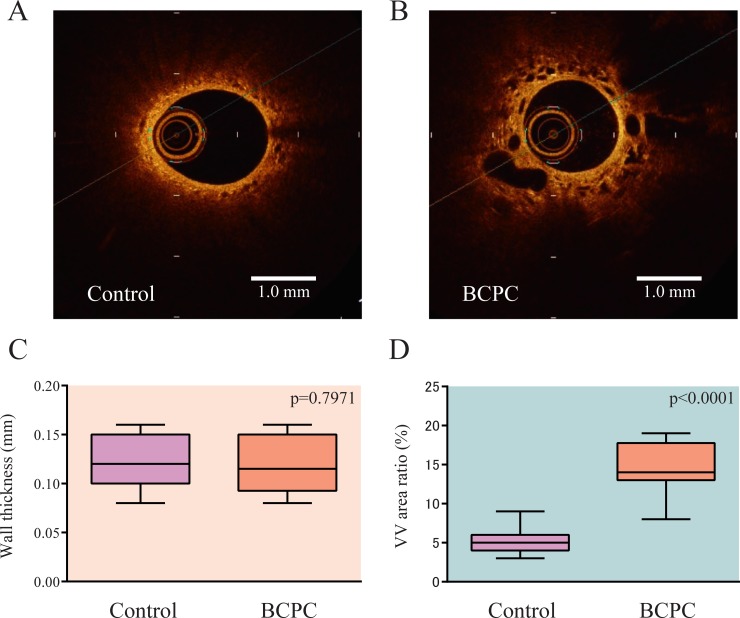
Representative images of pulmonary artery morphology as seen on OCT. Pulmonary artery walls of children in the control group (A) and in the BCPC group (B) are shown. The variously shaped lacunae represent the vasa vasorum (VV). Wall thickness was not significantly different between the two groups (C), although the VV area ratio was significantly increased in the BCPC group compared to the Control group (D). Boxes represent the interquartile range (IQR); central line: median; whiskers: 5th–95th percentiles; BCPC: bidirectional cavopulmonary connection.

Table 1 shows the clinical and hemodynamic data of the participants. Representative cross-sectional images of OCT are shown in Fig 1A and 1B. There was no significant difference in PA wall thickness between the BCPC and Control groups (0.12 ± 0.03 mm vs. 0.12 ± 0.02 mm, respectively) (Fig 1C). The VV area ratio was significantly greater in the BCPC group than in the Control group (14.5 ± 3.5% vs. 5.3 ± 1.6%, respectively; p<0.0001) (Fig 1D). The averages and variances of 5 cross sections measured in each patient are shown in Table 2.

**Table 1:** Subjects’ clinical characteristics.

	Control (n = 20)	BCPC (n = 8)	p value
Sex (male/female)	9/11	4/4	0.878
Age (y)	1.4 ± 0.4 (1.0–2.0)	1.3 ± 0.3 (1.1–1.9)	0.821.
Weight (kg)	10.5 ± 1.6 (8.0–13.1)	10.2 ± 1.1 (9.0–12.0)	0.797
Height (cm)	79.1 ± 4.1 (74.0–88.0)	78.5 ± 1.1 (76.9–81.0)	0.877
Heart rate (bpm)	111 ± 15 (89–129)	106 ± 10 (97–124)	0.745
Systolic blood pressure (mmHg)	82 ± 14 (67–115)	77 ± 9 (68–91)	0.541.
Diastolic blood pressure (mmHg)	42 ± 5 (37–51)	42 ± 5 (34–47)	0.928
mPAP (mmHg)	12.5 ± 3.3 (8–17)	10.4 ± 2.6 (8–13)	0.041
SaO2 (%)	99 ± 1 (97–100)	82 ± 4 (77–87)	< 0.0001
Time after BCPC (months)		7.1 ± 2.2 (4.0–11.2)	
Treatment		Furosemide 4 Spironolactone 4 Macitentan 4 HOT 5	

Data are shown as means ± SD and range in parentheses.

BCPC, bidirectional cavopulmonary connection; HOT, home oxygen therapy; mPAP, mean pulmonary arterial pressure; SaO2, arterial oxygen saturation

**Table 2:** The data of individual participants.

Control	Wall thickness (mm)	VV area ratio (%)
No. 1	0.08 ± 0.02	3.10 ± 0.14
2	0.10 ± 0.01	4.90 ± 0.27
3	0.11 ± 0.02	5.02 ± 0.23
4	0.09 ± 0.01	5.90 ± 0.28
5	0.12 ± 0.02	5.96 ± 0.80
6	0.15 ± 0.01	2.60 ± 0.56
7	0.16 ± 0.04	4.08 ± 0.35
8	0.15 ± 0.04	3.20 ± 0.25
9	0.12 ± 0.03	4.94 ± 0.79
10	0.15 ± 0.04	3.10 ± 0.41
11	0.16 ± 0.01	2.02 ± 1.43
12	0.15 ± 0.01	5.50 ± 0.16
13	0.14 ± 0.01	6.70 ± 0.36
14	0.11 ± 0.01	1.08 ± 0.30
15	0.09 ± 0.03	2.18 ± 0.72
16	0.12 ± 0.01	3.04 ± 1.53
17	0.09 ± 0.03	5.06 ± 1.11
18	0.10 ± 0.02	8.24 ± 2.36
19	0.15 ± 0.03	3.76 ± 0.37
20	0.14 ± 0.02	3.02 ± 0.13
**BCPC**		
No. 1	0.10 ± 0.01	5.10 ± 2.12
2	0.08 ± 0.01	8.64 ± 1.04
3	0.09 ± 0.01	9.12 ± 1.55
4	0.11 ± 0.02	13.84 ± 2.41
5	0.12 ± 0.01	11.06 ± 1.82
6	0.16 ± 0.03	15.72 ± 2.15
7	0.15 ± 0.03	14.10 ± 2.90
8	0.15 ± 0.04	18.40 ± 4.61

Data are shown as means ± SD of 5 cross-sections in each patient

BCPC, bidirectional cavopulmonary connection

## Discussion

This report indicated that OCT is an adequate imaging procedure for demonstration of the development of PA VV in patients after BCPC. Although the precise cellular and molecular mechanisms regulating adventitial neovascularization remain unknown, it has been demonstrated that hypoxia induces the development of PA VV, which form the microcirculatory network of the systemic circulation [1,2,4]. Vascular diseases are frequently associated with prominent expansion of the VV, which has led to the hypothesis that VV contribute to vascular remodeling by acting as a conduit for the delivery of leukocytes and progenitor cells [1,2,10]. Ours is the first report to quantify in vivo vessel anastomoses, microvasculature, and VV neovascularization in the PA circulation in patients after BCPC.

The results of this study indicate that visualization of the PAs using OCT can be clinically useful to assess pathological remodeling and therapeutic effects in patients after BCPC. This method might also find application in patients with other kinds of cyanotic congenital heart diseases, to predict the future quality of the circulatory condition and pulmonary vascular function.

### Limitations

In the present study, development of the VV was evaluated by the VV area ratio, defined as the VV area divided by the adventitial area in cross-sectional images. We defined the outer boundary of the adventitia as 500 μm outside the intima-media layer, because it is morphologically unclear. It would be necessary to determine the more appropriate measuring method in future. This study only demonstrated the enhanced presence of VV in the adventitia of the PA after the BCPC procedure, but did not assess its clinical significance. The correlation between the VV area ratio and SPCA flow volume should be evaluated in future to establish the clinical significance of PA OCT imaging in this patient group. It is also desirable to assess the relationship between the development of VV and oxygen saturation levels. The adventitia and VV may serve as a rich source of various cell types (resident fibroblast, myofibroblast, and local and circulating progenitor cells) that contribute to pathophysiological changes in vascular structure. Evaluation of sequential morphological changes in the VV after Fontan operation should also be conducted to assess future prognosis in these cases.

## Conclusion

Development of VV in children after BCPC can be evaluated using OCT. This technique might also be a promising new tool for evaluating PA remodeling in cyanotic congenital heart disease patients.

## Supporting information

S1 Table Measurement of PA wall thickness in each case. (XLSX)

S2 Table Measurement of VV area ratio in each case. (XLSX)
